# Laser photobiomodulation enhances cell viability and regenerative gene expression in oxidative-stressed muscle cells

**DOI:** 10.1007/s10103-026-04811-w

**Published:** 2026-02-05

**Authors:** Ana Flávia Spadaccini Silva-de-Oliveira, Jéssica Lúcio da Silva, Nathalia Thalitha Bernardes dos Santos, Rodrigo Antonio Carvalho Andraus, Regina Célia Poli-Frederico, Deise Aparecida de Almeida Pires Oliveira, Rodrigo Franco de Oliveira, Luciana Prado Maia

**Affiliations:** 1 UNIFIO University Center, Ourinhos, Brazil; 2 Graduate Program Associated in Rehabilitation Science UEL/UNOPAR, University Pitagoras Unopar Anhanguera, Londrina, Brazil; 3 Graduate Program in Human Movement and Rehabilitation, Evangelical University of Goias, Anápolis, Brazil; 4 Graduate Program in Dentistry, Evangelical University of Goias, Anapolis, Brazil

**Keywords:** Photobiomodulation, Laser therapy, Oxidative stress, Myoblasts

## Abstract

This study aimed to evaluate the effects of laser photobiomodulation therapy (LPT) on muscle cells subjected to oxidative stress. The primary objective was to determine whether LPT could preserve cell viability and modulate the expression of genes associated with muscle regeneration, specifically MyoD and *myogenin*, as well as the pro-inflammatory cytokine IL-6. Methods: C2C12 myoblasts were cultured and exposed to oxidative stress using hydrogen peroxide (H₂O₂) at a concentration of 50 µM for 1 h. Cells were then irradiated with LPT at wavelengths of 660–808 nm with fluences of 3, 5, and 10 J, applied either before (PRE-OS) or after (POST-OS) oxidative stress induction. Cell viability was assessed by the MTT assay, and gene expression was quantified using RT-qPCR. Results: Oxidative stress significantly reduced cell viability. LPT applied prior to OS with 660 nm (3 J) and 808 nm (3 and 10 J) attenuated this reduction. Notably, 10 J at 808 nm (PRE-OS) increased viability beyond control levels and markedly upregulated MyoD expression. *Myogenin* expression was also observed under 10 J PRE-OS conditions, while IL-6 expression was detected with 5 J at both wavelengths in PRE- and POST-OS groups. Conclusion: LPT demonstrated protective and regenerative effects on myoblasts under oxidative stress, preserving viability and enhancing regenerative gene expression. These findings support the potential of LPT as a therapeutic strategy for muscle injuries and disorders associated with oxidative stress.

## Introduction

Muscle injuries are highly prevalent in both sports and occupational settings, with musculoskeletal conditions accounting for more than 30% of injuries treated in medical clinics. These injuries may result from various mechanisms, including direct (e.g., lacerations or contusions) or indirect trauma (e.g., ischemia, loss of innervation, or excessive tension). Despite the diverse etiologies, the overall muscle repair is generally consistent across most cases [1].

Following an injury, the muscle repair process progresses through distinct yet interdependent phases. In the initial phase, muscle damage induces myofiber degeneration and necrosis, which in turn triggers an inflammatory response at the injury site, typically within 1 to 5 days post-injury. Between 5 and 9 days after the injury, muscle progenitor cells are activated and initiate the regeneration process, fusing with damaged fibers and with each other to form new myofibers [2].

The inflammatory phase of muscle healing is characterized by elevated production of reactive oxygen species (ROS) and reactive nitrogen species (RNS), accompanied by reduced activity of antioxidant defense enzymes. This imbalance between pro-oxidants and antioxidants leads to oxidative and nitrosative stress. When produced in excess, these reactive species can impair muscle repair by exacerbating inflammation and inhibiting myoblast differentiation, ultimately compromising muscle tissue regeneration [2].

In the context of muscle injury and oxidative stress, interleukin-6 (IL-6), produced by both immune and muscle cells, is released early and functions as a myokine that promotes satellite-cell proliferation and myogenic differentiation under acute conditions. However, when chronically elevated, IL-6 is associated with muscle wasting and impaired regeneration [3–5]. Following the activation of myogenic cells, MyoD is rapidly expressed, and plays a pivotal role in myogenesis, regulating the proliferation and differentiation of precursor cells into myoblasts [6]. Thus, increased MyoD expression serves as a marker of the initiation of the regenerative process [7]. In contrast, *myogenin* governs the terminal differentiation of myoblasts into mature myotubes and the fusion process required for new fiber formation, making it a key indicator of the later stages of muscle regeneration [6, 8]. Taken together, modulation of IL-6, along with enhanced MyoD and *myogenin* expression, may provide mechanistic support for therapeutic interventions aimed at promoting muscle repair under oxidative stress conditions.

There is increasing evidence that reactive oxygen species (ROS), including free radicals and hydrogen peroxide (H₂O₂), contribute to the pathophysiology of neurodegenerative diseases. H₂O₂ is considered one of the main agents responsible for oxidative modifications and damage to macromolecules such as DNA, thereby disrupting normal cellular functions and compromising cellular integrity. Therefore, maintaining a balance between oxidants and antioxidants is crucial to protecting against - or contributing to - cellular degeneration [9].

Given the importance of the muscle healing processes, substances or therapies that promote repair and enhance antioxidant defenses are essential for maintaining low levels of free radical production and minimizing factors that may delay or impair tissue recovery [10]. Among the therapies investigated, laser photobiomodulation therapy (LPT) stands out. It employs monochromatic light in the red or infrared spectrum to treat various tissues in a non-destructive and non-thermal manner. The therapeutic effect is based on the ability of light to modulate cellular metabolism, particularly through its absorption by mitochondria and cytochrome C oxidase [11].

Several laser properties determine its therapeutic applicability, including continuous or pulsed wave emission, intensity, monochromaticity, and collimation or directionality. These properties are fundamental for low-intensity therapies, which act through the excitation of endogenous chromophores in biological tissues. Such chromophores absorb visible (red) or invisible (infrared) radiation at various doses without causing tissue heating [12].

LPT applied either before or after an injury has shown beneficial and protective effects on muscle repair. These include modulation of the inflammatory response, promotion of angiogenesis, remodeling of collagen fibers, and formation of new muscle fibers [2]. However, considerable variability and inconclusive treatment parameters have been reported in the literature. Moreover, while some studies have investigated the generation of reactive oxygen species (ROS) by LPT in cell cultures [12–14], they have not specifically addressed its antioxidant effects.

Therefore, the aim of this study was to evaluate the effects of LPT on cell viability, as well as on the expression of growth factors and pro-inflammatory cytokines, in muscle cells subjected to an oxidative stress protocol.

## Methods

### Cell culture

C2C12 myoblasts (Mus Musculus – Rio de Janeiro Cell Bank) were cultured in 75 cm³ flasks, with Dulbecco’s Modified Eagle’s Medium (DMEM - Gibco™ - Invitrogen Corporation, Grand Island, USA), supplemented with 10% fetal bovine serum (Gibco™) and 1% antibiotic-antimycotic solution (Gibco™). The cells were maintained in an incubator at 37 °C in a humidified atmosphere with 5% CO_2_. Upon reaching 80% semi-confluence, they were transferred to 96-well plates (100 mm² - TPP, Switzerland, Europe) at a density of 1 × 10^4^ cells/ml and incubated for 24 h to allow sedimentation before treatment.

## Oxidative stress protocol

To establish the concentration of hydrogen peroxide capable of inducing deleterious effects on cell viability, cells were treated with H_2_O_2_ at concentrations of 10, 50, 100 and 150 µM after 24 h of sedimentation. Cell viability was assessed at 1 and 4 h post-treatment using the mitochondrial tetrazolium test (MTT) assay.

## LPT treatment

Myoblasts subjected to oxidative stress with H₂O₂ at the optimal concentration were treated with LPT. The laser device used was the Laser Therapy EC (Thera Laser DMC Equipment Ltda, Brazil), which was fixed on a support, while the plate was positioned to direct irradiation toward the wells. Cells were plated with spaces between wells, and Teflon separators were placed in empty wells to prevent energy absorption in adjacent wells by reflection [15]. This material was chosen due to its combination of chemical, thermal, and mechanical stability, high fractional free volume, low refractive index, low surface energy, and broad optical transparency [16]. The plate lid was also covered with a black film to ensure that the irradiation was directed specifically to the treatment well.

The parameters used are summarized in Table 1, which presents fluences of 3, 5, and 10 J at wavelengths of 660 and 808 nm, applied either before (Pre-OS) or 1 h after (Post-OS) oxidative stress. Cells cultured in standard medium served as the negative control, whereas cells subjected to oxidative stress without irradiation served as the positive control. The experimental groups were defined as described in Table 2.

The C2C12 cell line differentiates rapidly, forming contractile myotubes. Consequently, only one application of LPT was performed, and analyses were conducted 24 h after treatment.

**Table 1:** Irradiation parameters

Target beam spot size (cm^2^)	0.0324
Irradiance (mW/cm^2^)	3086.4
Duration of exposure (s)	30–50 – 100
Fluence or energy density (J/cm^2^)	9.09–15.15–30.30
Energy delivered (J)	3–5 – 10
Number of irradiated points	1
Irradiated area or area of the laser tip (cm²)	0.33
Distance from the laser tip (cm)	1
Number and frequency of treatment session	1 application (before or after 24 h after OS)
Total energy delivered (J)	3–5 – 10

**Table 2:** Experimental groups, treatments, and study design

Group	Condition / Treatment	Laser Parameters	Timing of Irradiation	Description
**C**	Negative control	—	—	Untreated cells (no H₂O₂ or LPT)
**OS**	Positive control	—	—	Cells treated with H₂O₂ (50 µM for 1 h) without irradiation
**3J_660nm_Pre-OS**	Oxidative stress + LPT	660 nm, 3 J	Before OS induction	Cells irradiated before H₂O₂ exposure
**5J_660nm_Pre-OS**	Oxidative stress + LPT	660 nm, 5 J	Before OS induction	Cells irradiated before H₂O₂ exposure
**10J_660nm_Pre-OS**	Oxidative stress + LPT	660 nm, 10 J	Before OS induction	Cells irradiated before H₂O₂ exposure
**3J_660nm_Post-OS**	Oxidative stress + LPT	660 nm, 3 J	1 h after OS induction	Cells irradiated after H₂O₂ exposure
**5J_660nm_Post-OS**	Oxidative stress + LPT	660 nm, 5 J	1 h after OS induction	Cells irradiated after H₂O₂ exposure
**10J_660nm_Post-OS**	Oxidative stress + LPT	660 nm, 10 J	1 h after OS induction	Cells irradiated after H₂O₂ exposure
**3J_808nm_Pre-OS**	Oxidative stress + LPT	808 nm, 3 J	Before OS induction	Cells irradiated before H₂O₂ exposure
**5J_808nm_Pre-OS**	Oxidative stress + LPT	808 nm, 5 J	Before OS induction	Cells irradiated before H₂O₂ exposure
**10J_808nm_Pre-OS**	Oxidative stress + LPT	808 nm, 10 J	Before OS induction	Cells irradiated before H₂O₂ exposure
**3J_808nm_Post-OS**	Oxidative stress + LPT	808 nm, 3 J	1 h after OS induction	Cells irradiated after H₂O₂ exposure
**5J_808nm_Post-OS**	Oxidative stress + LPT	808 nm, 5 J	1 h after OS induction	Cells irradiated after H₂O₂ exposure
**10J_808nm_Post-OS**	Oxidative stress + LPT	808 nm, 10 J	1 h after OS induction	Cells irradiated after H₂O₂ exposure

## Cell viability – MTT test

After 24 h of oxidation, cell viability was assessed using the MTT assay (Invitrogen™). Briefly, MTT solution (5 mg/mL) was added to the culture medium at a final concentration of 10%, and the cells were incubated at 37 °C in a humidified atmosphere with 5% CO₂ and 95% air for 4 h. The solution was then removed, and 100 µL of dimethyl sulfoxide (DMSO, Sigma-Aldrich, St. Louis, MO, USA) was added to each well. The plates were shaken for 10 min, and absorbance was measured at 570 nm using an ELISA reader (SpectraCount – Packard Instrument, Offenburg, Germany). Cell viability was calculated as a percentage relative to the negative control according to the following formula:

$$\:Cell\:viability\:\left(\%\right)=\left(\frac{ODt-ODb}{ODc-ODb}\right)*100$$

where OD = optical density; tt = treatment; bb = blank; cc = control. This experiment was performed in triplicate.

## Gene expression Quantification – RTq-PCR

After 24 h of the irradiation protocol, RNA was extracted from the cells and reverse-transcribed into cDNA using the Cells-to-cDNA™ II kit (Thermo Fisher), following the manufacturer’s instructions. Total RNA was quantified with a NanoDrop Lite^®^ spectrophotometer (Thermo Fisher). For PCR analysis, reactions were carried out using TaqMan probes (Thermo Fisher) targeting both the genes of interest and the reference genes, combined with Master Mix Standard (Thermo Fisher) and the samples. Amplification and detection were performed on a StepOne Plus platform (Applied Biosystems).

The reactions were carried out in a final volume of 20 µL, consisting of 10 µL of Master Mix Standard (Thermo Fisher), 8 µL of ultrapure H₂O, 1 µL of TaqMan probes (Thermo Fisher), and 1 µL of cDNA. Amplification was performed for 40 cycles under the following conditions: 2 min at 50 °C, 10 min at 95 °C, 15 s at 95 °C, and 1 min at 60 °C, with a total runtime of approximately 2 h. The GAPDH gene was used as the endogenous control.

To determine the relative expression of the genes, quantitative expression values for the target genes interleukin 6 (IL-6), myoblast determination protein 1 (MyoD), and *myogenin*, along with the reference gene glyceraldehyde-3-phosphate dehydrogenase (GAPDH), were obtained by analyzing the exponential phase of the amplification curve (threshold - Ct). The Cts for each sample were calculated relative to the genes, and relative quantification (QR) was determined using the equation proposed by Livak and Schmittgen [17] (QR = 2^–∆∆CT^). The experiment was conducted in duplicate. This methodological approach was deemed appropriate because RT-qPCR is a highly sensitive and reproducible technique, allowing for reliable quantification even with duplicate measurements. RNA extraction from individual wells was not feasible due to the limited amount of cellular material available in each well; therefore, the samples were pooled to obtain sufficient RNA yield for RT-qPCR analysis. Pooling was performed within each experimental group to maintain sample integrity and comparability.

### Statistical analysis

For data from the oxidative stress protocol and cell viability assays, normality was assessed using the Shapiro-Wilk test, followed by a two-way ANOVA for inter-group comparisons. Dunnett’s post hoc test was applied for comparisons with the control group, while Tukey’s post hoc test was used for other comparisons. A significance level of 5% was adopted. Data analyses were performed using GraphPad Prism 5.0. For gene expression quantification, descriptive analyses were conducted, since the experiment was conducted in duplicate.

## Results

### Oxidative stress protocol

**Fig. 1:**
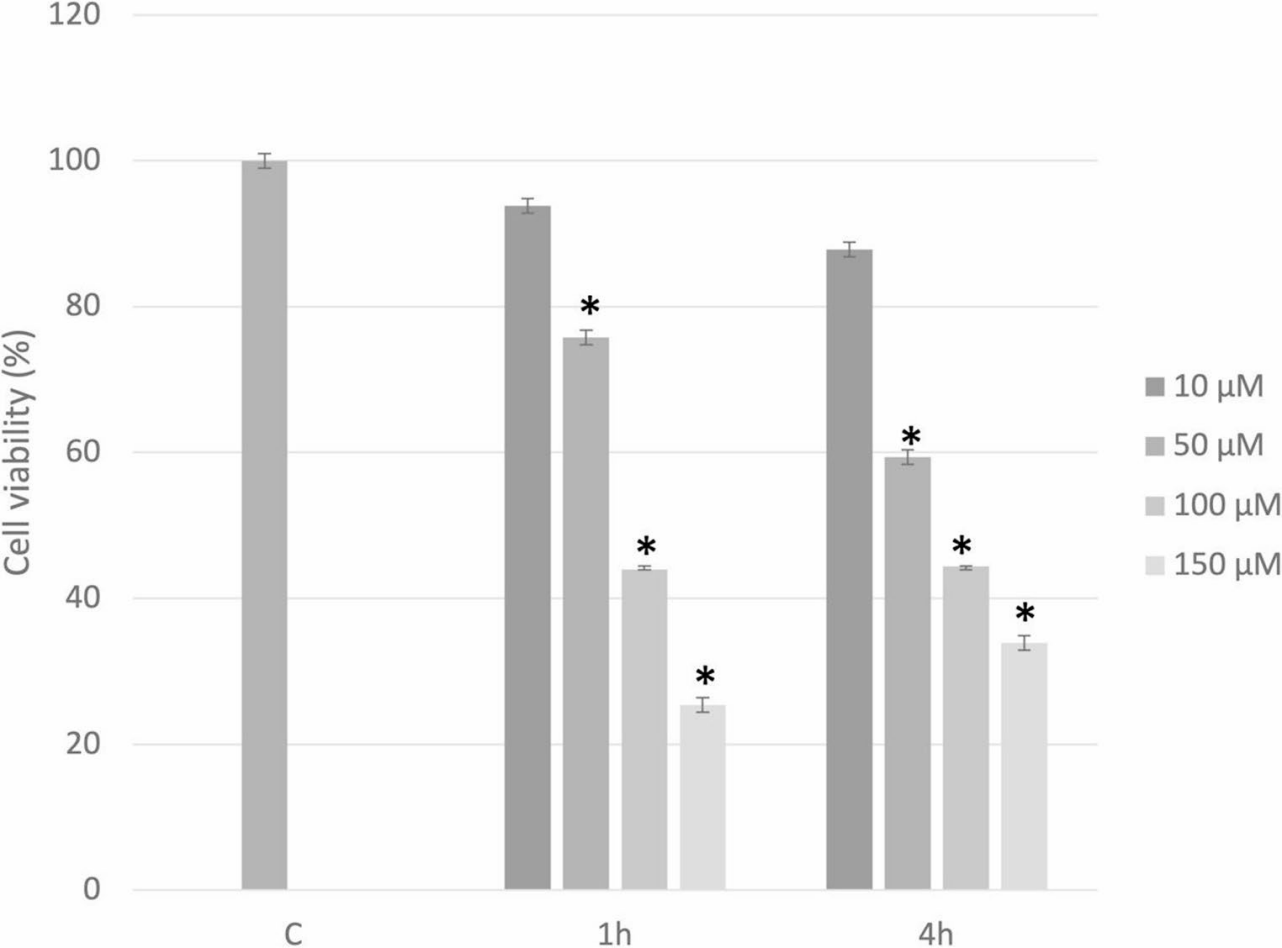
Mean cell viability (%) and standard deviation of C2C12 myoblasts treated with different concentrations of hydrogen peroxide for 1–4 h. * = statistically significant difference compared with the control group (C), two-way ANOVA (*p* < 0.05)

The effects of hydrogen peroxide at different concentrations on cell viability are shown in Fig. 1. Significant reductions compared with the control group were observed at 50 µM (*p* < 0.01), 100 µM (*p* < 0.01), and 150 µM (*p* < 0.01). Notably, 50 µM decreased cell viability by approximately 25% after 1 h of treatment. At higher concentrations, reductions exceeded 50%, which is undesirable, as extensive cell damage would compromise the effectiveness of LPT. Lower concentrations did not produce significant decreases in viability. Based on these results, it was determined that 1 h of treatment at 50 µM was sufficient to affect cell viability, and this concentration was selected for subsequent experiments.

### Cell viability - MTT

**Fig. 2:**
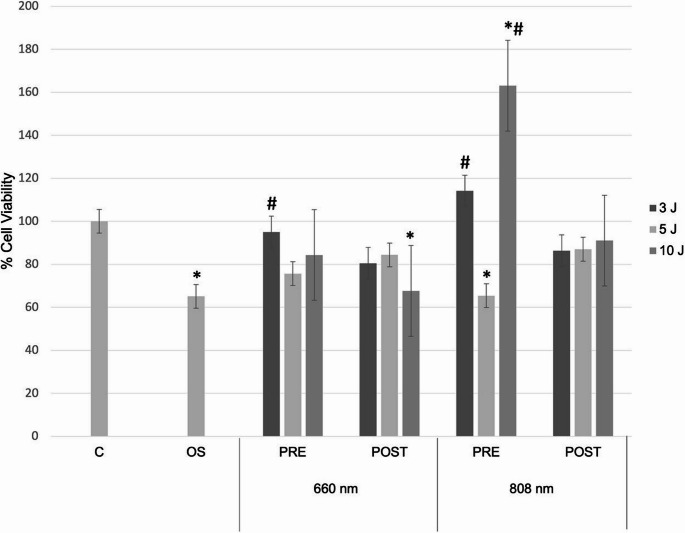
Mean cell viability (%) and standard deviation of C2C12 myoblasts subjected to oxidative stress (OS) and irradiated with different energies delivered and wavelengths, before (PRE) and after (POST) OS. * = statistically significant difference compared with the control group (C); # = statistically significant difference compared with the OS group; two-way ANOVA (*p* < 0.05)

Regarding LPT in C2C12 cells subjected to OS (Fig. 2), with exception of groups 660 nm 10 J POST (*p* = 0.064), 808 nm 5 J PRE (*p* = 0.094) and 808 nm 10 J PRE (*p* < 0.0001), all other treatments exhibited cell viability comparable to group C (*p* > 0.05), indicating that LPT attenuated the reduction in viability caused by OS. Additionally, groups 606 nm 3 J PRE and 808 nm 3 J PRE significantly increased cell viability compared to OS group (*p* = 0.0486 and *p* = 0,0003, respectively). Notably, 808 nm 10 J PRE group showed a significant increase in cell viability compared to both the C and OS groups (*p* < 0.0001).

### Genes quantification

**Fig. 3:**
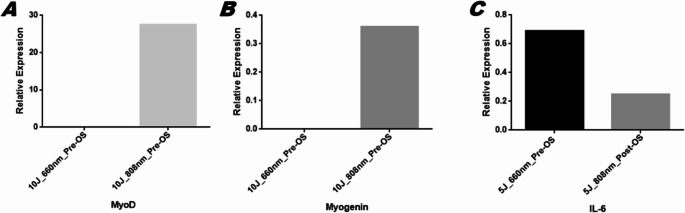
Relative quantification (QR = 2^–∆∆CT^) of MyoD, *myogenin* and IL-6 gene expression in C2C12 myoblasts subjected to oxidative stress and treated with laser photobiomodulation therapy. A: Expression of MyoD in groups irradiated with 10 J at 660 and 808 nm PRE. B: Expression of *myogenin* in groups irradiated with 10 J at 660 and 808 nm PRE. C: Expression of IL-6 in groups irradiated with 5 J at 660 nm PRE and 808 nm POST

Relative quantification analysis showed that the MyoD gene was expressed in the groups irradiated with 10 J at both 660 nm and 808 nm PRE (0.00 and 27.59, respectively). A similar pattern was observed for *myogenin* expression, with values of 0.00 at 660 nm and 0.36 at 808 nm. Interleukin-6 (IL-6) expression was detected in the groups irradiated with 5 J at 660 nm PRE and 808 nm POST, with values of 0.69 and 0.25, respectively. Notably, a significant increase in MyoD relative expression was observed exclusively in the group irradiated with 10 J at 808 nm PRE (Fig. 3). As no gene expression was detected in the remaining experimental groups, these results were omitted from the analysis for clarity.

## Discussion

This study aimed to evaluate the effects of LPT on muscle cells subjected to oxidative stress by assessing cell viability and the expression of genes associated with muscle regeneration, as well as a pro-inflammatory cytokine. To date, no studies in the literature have investigated this specific protocol of LPT in myoblasts under oxidative stress in vitro. However, several in vivo studies have examined muscle tissue regeneration following injury, demonstrating reductions in inflammatory markers and oxidative stress, particularly in relation to physical activity.

In our study, oxidative stress led to a significant reduction in cell viability after 1 h of treatment with 50 µM H_2_O_2_. Higher concentrations of H_2_O_2_ induced reductions exceeding 50% in cell viability, highlighting their detrimental effects on muscle cells.

The adopted protocol allowed the evaluation of myoblast behavior when irradiated with LPT either before or after exposure to oxidative stress. Myoblasts treated with LPT at 660 nm with an energy delivered of 3 J, and at 808 nm with energies of 3 and 10 J, prior to oxidative stress induction, exhibited attenuation of the reduction in cell viability. Except for groups irradiated at 660 nm with 10 J after oxidative stress, and at 808 nm with 5 J and 10 J before oxidative stress, all other treatments showed cell viability levels comparable to the control group, indicating that LPT mitigated the deleterious effects of oxidative stress.

One study [18] also induced oxidative stress using a different protocol, evaluating the effects of LPT on C2C12 myoblasts cultured in various concentrations of M1 phenotype macrophage-conditioned medium (MCM1) derived from J774 cells. In that study, irradiation was performed 2 h after the oxidative stress protocol. The authors reported that LPT effectively modulated the increase in viability and proliferation of myoblasts when irradiated with low-level laser at a wavelength of 780 nm and an energy delivered of 1 J. In our findings, LPT applied after oxidative stress also influenced cell viability, although at higher energies delivered − 3 and 5 J at 660 nm, and 3, 5, and 10 J at 808 nm - since the viability of these groups was comparable to that of the control group.

Studies analyzing myoblasts have also reported favorable outcomes regarding cell viability. Trajano et al. [19] investigated C2C12 myoblast cultures exposed to low-level infrared laser (808 nm, 100 mW) at fluences of 10, 35, and 70 J/cm^2^, with evaluations at 24, 48, and 72 h. Their findings showed that laser exposure enhanced cell viability after 48 h with a fluence of 10 J/cm^2^, whereas no significant increase was observed at higher fluences. These results are consistent with our study, in which LPT improved myoblast viability even under oxidative stress, particularly with the energy delivered of 10 J at 808 nm.

Similarly, using the infrared spectrum, Mesquita-Ferrari et al. [20] irradiated C2C12 cells with a GaAlAs diode laser at a wavelength of 780 nm and an energy delivered of 5 J. Cell viability and the expression of myogenic regulatory factors were assessed at 24, 48, and 72 h post-irradiation using the MTT assay and quantitative RT-qPCR, respectively. Their results showed increased cell viability in the laser-treated group compared to the control at all time points. In our study, which included oxidative stress, application of 5 J at 660 nm either before or after oxidative stress did not result in significant differences compared with the control and OS groups. These findings suggest that the presence of oxidative stress may alter the cellular response to LPT, as the energy delivered of 5 J at 660 nm did not reproduce the viability gains observed in previous studies, underscoring the importance of experimental conditions when evaluating the biological effects of laser therapy.

Regarding the gene expression results of the present study, increased levels of MyoD and *myogenin* were observed in the groups irradiated with 10 J at both wavelengths in the PRE-OS condition, with MyoD showing the most pronounced expression at 10 J with 808 nm. In addition, IL-6 expression was detected at the energy delivered of 5 J, with the 660 nm wavelength showing expression in the PRE and the 808 nm wavelength in the POST-OS condition. However, in the study by Mesquita-Ferrari et al. [20] using an energy delivered of 5 J, a trend toward increased *myogenin* messenger RNA (mRNA) was observed in the laser-treated group. Conversely, and in agreement with our findings, Trajano et al. [13] reported an increase in MyoD expression in C2C12 myoblasts treated with a low-level infrared laser at an energy delivered of 10 J.

 In vivo studies have also been conducted using both animal and human models. For instance, Alves et al. [9]. demonstrated that low-level laser therapy (LLLT) administered at 780 nm and an energy delivered of 10 J following muscle injury modulated the expression of MyoD and *myogenin* during the repair process. Similarly, Rodrigues [21], using LPT at 660 nm with energies delivered of 10 and 50 J, observed upregulation of MyoD at 50 J and increased *myogenin* expression at 10 J. In contrast, our study showed expression of MyoD and *myogenin* in groups irradiated prior to the oxidative stress protocol, differing from these studies in which LPT was applied after the injury.

In the present study, the best results were obtained with the enegy delivered of 10 J at 808 nm in the PRE-OS protocol. This group showed a significant increase in cell viability compared to the controls, as well as a higher relative expression of the MyoD gene, indicating enhanced cell regeneration. These findings are consistent with clinical studies. For example, de Oliveira et al. [22], applied LPT at 10 J and 810 nm prior to a fatigue protocol and analyzed inflammatory markers related to fatigue and oxidative stress at 1 min, 1 h, 24 h, 48 h, 72 h, and 96 h post-treatment. They observed a reduction in these markers, which favored muscle performance. Similarly, Vanin et al. [23] investigated LPT administered before an eccentric exercise protocol using energies delivered of 10, 30, and 50 J at 810 nm on the quadriceps, with evaluations conducted at 1, 24, 48, 72, and 96 h. Their results demonstrated that laser treatment enhanced maximum voluntary contraction from 24 to 96 h post-application at enegies delivered of 10 J and 50 J, thereby improving performance and favorably modulating biological markers such as CK and IL-6.

It is well established that the effectiveness of LPT on target tissues depends on a combination of parameters, including wavelength, energy density, duration, and frequency of application. Among these, wavelength plays a pivotal role in laser-tissue interactions by modulating absorption and scattering properties [24]. Given the wide range of laser parameters and the comprehensive protocol employed in this study, we were unable to evaluate additional doses. Further studies are needed to investigate the effects of LPT under oxidative stress, optimize its application for improving muscle function, and clarify the mechanisms underlying its protective effects, which may contribute to the development of therapeutic strategies for muscle injuries and disorders associated with oxidative stress.

## Conclusion

The present study demonstrates that oxidative stress significantly compromises muscle cell viability. However, the application of LPT emerges as a promising strategy to counteract these effects. Specifically, irradiation at 808 nm with 10 J, applied prior to oxidative stress induction, not only preserved cell viability, but also enhanced the expression of key genes involved in muscle regeneration, such as MyoD and *myogenin*. These findings suggest that LPT may play a critical role in promoting muscle cell recovery and regeneration under oxidative stress.

## Data Availability

No datasets were generated or analysed during the current study.

## References

[1] dos Santos SA, Serra AJ, Stancker TG et al (2017) Effects of photobiomodulation therapy on oxidative stress in muscle injury animal models: A systematic review. Oxid Med Cell Longev 2017:5273403. 10.1155/2017/5273403 29075364 10.1155/2017/5273403 PMC5623775

[2] Tu H, Li YL (2023) Inflammation balance in skeletal muscle damage and repair. Front Immunol. 26;14:1133355. 10.3389/fimmu.2023.1133355 10.3389/fimmu.2023.1133355 PMC9909416 36776867

[3] Steyn PJ, Dzobo K, Smith RI, Myburgh KH (2019) Interleukin-6 induces myogenic differentiation via JAK2-STAT3 signaling in mouse C2C12 myoblast cell line and primary human myoblasts. Int J Mol Sci 20(21):5273. 10.3390/ijms20215273 31652937 10.3390/ijms20215273 PMC6862063

[4] Muñoz-Cánoves P, Scheele C, Pedersen BK, Serrano AL (2013) Interleukine-6 myokine signaling in skeletal muscle: a double-edged sword? FEBS J 280(17):4131–4148 23663276 10.1111/febs.12338 PMC4163639

[5] Serrano AL, Baeza-Raja B, Perdiguero E, Jardi M, Muñoz-Cánoves P (2008) Interleukin-6 is an essential regulator of satellite Cell-Mediated skeletal muscle hypertrophy. Cell Metab 7(1):33–44. 10.1016/j.cmet.2007.11.011 18177723 10.1016/j.cmet.2007.11.011

[6] Benavente-Diaz M, Comai G, Di Girolamo D et al (2021) Dynamics of myogenic differentiation using a novel Myogenin knock-in reporter mouse. Skelet Muscle 11:5. 10.1186/s13395-021-00260-x 33602287 10.1186/s13395-021-00260-x PMC7890983

[7] Zammit PS (2017) Function of the myogenic regulatory factors Myf5, MyoD, Myogenin and MRF4 in skeletal muscle, satellite cells and regenerative myogenesis. Semin Cell Dev Biol 72:19–32. 10.1016/j.semcdb.2017.11.011 29127046 10.1016/j.semcdb.2017.11.011

[8] Hernández-Hernández JM, García-González EG, Brun CE, Rudnicki M (2017) The myogenic regulatory Factors, determinants of muscle Development, cell identity and regeneration. Semin Cell Dev Biol 15(72):10–18. 10.1016/j.semcdb.2017.11.010 10.1016/j.semcdb.2017.11.010 PMC5723221 29127045

[9] Alves AN, Ribeiro BG, Fernandes KPS et al (2016) Comparative effects of low-level laser therapy pre- and post-injury on mRNA expression of MyoD, myogenin, and IL-6 during the skeletal muscle repair. Lasers Med Sci 31:679–685. 10.1007/s10103-016-1908-9 26914683 10.1007/s10103-016-1908-9

[10] Wang JY, Huang ZQ, Deng HP, Zhao L, Deng HY, Liu JP, Shen XY, Cheng K (2022) Low level light therapy/photobiomodulation for diabetic peripheral neuropathy: protocol of a systematic review and meta-analysis. BMJ Open 14;12(9):e059476 10.1136/bmjopen-2021-059476 10.1136/bmjopen-2021-059476 PMC9476114 36104132

[11] Marrow B, Stem S, Conditioned C et al (2019) Protective effect of photobiomodulation therapy media on pheochromocytoma cell line 12 against. Laser Appl Med Sci Res Cent 10:163–170. 10.15171/jlms.2019.26 10.15171/jlms.2019.26 PMC6817795 31749940

[12] 8 de Souza Da, Fonseca A, Presta GA, Geller M et al (2012) Low-intensity infrared laser increases plasma proteins and induces oxidative stress in vitro. Lasers Med Sci 27:211–217. 10.1007/s10103-011-0945-7 21701880 10.1007/s10103-011-0945-7

[13] 9 Trajano LASN, Stumbo AC, da Silva CL et al (2016) Low-level infrared laser modulates muscle repair and chromosome stabilization genes in myoblasts. Lasers Med Sci 31:1161–1167. 10.1007/s10103-016-1956-1 27220530 10.1007/s10103-016-1956-1

[14] 10 Macedo AB, Moraes LHR, Mizobuti DS et al (2015) Low-Level laser therapy (LLLT) in dystrophin-deficient muscle cells: effects on regeneration capacity, inflammation response and oxidative stress. PLoS ONE 10:1–14. 10.1371/journal.pone.0128567 10.1371/journal.pone.0128567 PMC4470633 26083527

[15] Heymann PGB, Ziebart T, Kämmerer PW et al (2016) The enhancing effect of a laser photochemotherapy with cisplatin or zolendronic acid in primary human osteoblasts and osteosarcoma cells in vitro. J Oral Pathol Med 45:803–809. 10.1111/jop.12442 27122094 10.1111/jop.12442

[16] Zhang H, Weber SG (2011) Teflon AF materials. Top Curr Chem 308:307–338. 10.1007/128 10.1007/128_2011_249 21935770

[17] Livak KJ, Schmittgen TD (2001) Analysis of relative gene expression data using real-time quantitative PCR and the 2-∆∆CT method. Methods 25:402–408. 10.1006/meth.2001.1262 11846609 10.1006/meth.2001.1262

[18] dos Santos TC, de Brito Sousa K, Andreo L et al (2020) Effect of photobiomodulation on C2C12 myoblasts cultivated in M1 Macrophage-conditioned media. Photochem Photobiol 96:906–916. 10.1111/php.13215 31907936 10.1111/php.13215

[19] Trajano ASN, da Silva CL, de Carvalho SN et al (2016) Cell viability, reactive oxygen species, apoptosis, and necrosis in myoblast cultures exposed to low-level infrared laser. Lasers Med Sci 31:841–848. 10.1007/s10103-016-1909-8 26886589 10.1007/s10103-016-1909-8

[20] Mesquita-Ferrari RA, Alves AN, de Oliveira Cardoso V et al (2015) Low-level laser irradiation modulates cell viability and creatine kinase activity in C2C12 muscle cells during the differentiation process. Lasers Med Sci 30:2209–2213. 10.1007/s10103-015-1715-8 25616713 10.1007/s10103-015-1715-8

[21] Rodrigues NC, Brunelli R, De Araújo HSS et al (2013) Low-level laser therapy (LLLT) (660 nm) alters gene expression during muscle healing in rats. J Photochem Photobiol B Biol 120:29–35. 10.1016/j.jphotobiol.2013.01.002 10.1016/j.jphotobiol.2013.01.002 23416710

[22] de Oliveira AR, Tomazoni SS, de Carvalho P, de TC et al (2017) Pre-Exercise infrared photobiomodulation therapy (810 nm) in skeletal muscle performance and postexercise recovery in humans: what is the optimal power output? Photomed Laser Surg 35:595–603. 10.1089/pho.2017.4343 29099680 10.1089/pho.2017.4343

[23] Vanin AA, De Marchi T, Tomazoni SS et al (2016) Pre-Exercise infrared Low-Level laser therapy (810 nm) in skeletal muscle performance and postexercise recovery in Humans, what is the optimal dose? A Randomized, Double-Blind, Placebo-Controlled clinical trial. Photomed Laser Surg 34:473–482. 10.1089/pho.2015.3992 27575834 10.1089/pho.2015.3992

[24] Kocherova I, Bryja A, Błochowiak K et al (2021) Photobiomodulation with red and near-infrared light improves viability and modulates expression of mesenchymal and apoptotic-related markers in human gingival fibroblasts. Mater (Basel) 14. 10.3390/ma14123427 10.3390/ma14123427 PMC8233986 34205573

